# Effect of an Intravenous Acetaminophen/Ibuprofen Fixed-Dose Combination on Catheter-Related Bladder Discomfort: A Prospective, Randomized, Placebo-Controlled, Double-Blind Pilot Study

**DOI:** 10.3390/medicina62061038

**Published:** 2026-05-27

**Authors:** Hwang-Ju You, Ji-Yoon Jung, Woojin Kwon, Sung-Ae Cho, Tae-Yun Sung

**Affiliations:** 1 Department of Anesthesiology and Pain Medicine, Konyang University Hospital, Konyang University College of Medicine, Daejeon 35365, Republic of Korea; 200767@kyuh.ac.kr (H.-J.Y.); 200756@kyuh.ac.kr (J.-Y.J.); 200723@kyuh.ac.kr (W.K.); 2 Department of Anesthesiology and Pain Medicine, Samsung ChangWon Hospital, Sungkyunkwan University School of Medicine, ChangWon 51353, Republic of Korea; sachosa@naver.com

**Keywords:** urinary catheterization, catheter-related bladder discomfort, urological surgical procedure, acetaminophen, ibuprofen

## Abstract

*Background and Objectives*: Catheter-related bladder discomfort (CRBD) commonly arises as a direct consequence of perioperative urinary catheterization. A fixed-dose combination of 1000 mg acetaminophen and 300 mg ibuprofen provides multimodal analgesia. In this study, we assessed the impact of this fixed-dose combination on mitigating CRBD in patients undergoing urological procedures. *Materials and Methods*: In this prospective pilot study, 23 patients undergoing urological surgery requiring urinary catheterization were randomized into two groups; approximately 20 min before the anticipated end of surgery, patients were administered a combination of 1000 mg acetaminophen and 300 mg ibuprofen (intervention group, *n* = 11) or saline (control group, *n* = 12). The primary endpoint was the incidence of CRBD immediately after the patient’s arrival at the post-anesthetic care unit (PACU). The incidence of CRBD at 1, 2, and 6 h postoperatively and the severity of CRBD at each time point were also assessed. *Results*: The incidence of CRBD immediately after arrival at the PACU was lower in the intervention group (54.5% vs. 100%, *p* = 0.014). However, no significant differences in overall CRBD incidence were observed at later postoperative time points. The incidence of moderate CRBD was lower in the intervention group at 0 h and 1 h (*p* = 0.036 and 0.037, respectively). *Conclusions*: The findings of this pilot randomized trial provide preliminary evidence that intravenous acetaminophen and ibuprofen may reduce early postoperative CRBD following urological surgery. Given the small sample size and single-center design, larger multicenter randomized studies are needed to confirm these findings.

## 1. Introduction

Indwelling urinary catheterization is widely utilized in perioperative care, particularly for patients undergoing urological surgery. However, this intervention frequently triggers catheter-related bladder discomfort (CRBD), with reported incidences ranging from 47% to 90% following procedures [1,2]. Characterized by urinary urgency and suprapubic discomfort, CRBD is often poorly responsive to opioid analgesics and may contribute to postoperative complications, including emergence agitation, impaired quality of recovery, challenges in pain management, and risks of physical injury or secondary infection [3,4]. Consequently, uncontrolled CRBD may hinder Enhanced Recovery After Surgery (ERAS) pathways by delaying early ambulation and reducing overall patient satisfaction [4].

The pathophysiology of CRBD involves involuntary detrusor contractions mediated by muscarinic receptors, combined with prostaglandin-driven afferent sensitization resulting from local bladder irritation [5,6]. Maxigesic^®^ (Kyungbo Pharm, Asan, Republic of Korea), an intravenous fixed-dose combination of 1000 mg acetaminophen and 300 mg ibuprofen, was recently introduced in South Korea and provides multimodal analgesia through complementary central and peripheral mechanisms [7,8]. This fixed-dose combination has demonstrated favorable efficacy and safety in managing postoperative pain [9]. Ibuprofen inhibits prostaglandin-mediated inflammation, which contributes to bladder afferent sensitization, while acetaminophen modulates central nociceptive processing. This dual mechanism may be particularly effective for CRBD, which involves both peripheral irritation and enhanced afferent signaling. Given the absence of clinical evidence regarding this combination for CRBD, we hypothesized that the combined administration of acetaminophen and ibuprofen could reduce the incidence and severity of postoperative CRBD, particularly clinically significant moderate-to-severe symptoms requiring rescue treatment during the early postoperative period.

## 2. Materials and Methods

Following Institutional Review Board approval (KYUH 2022-04-007-001), this prospective randomized study was registered with the Korea Clinical Research Information Service (permit number: KCT0007640). This prospective, randomized, placebo-controlled study was conducted from December 2022 through September 2025 at a single university hospital after obtaining written informed consent from all participants. During the informed consent process, patients were educated about the typical symptoms of CRBD, including urinary urgency, suprapubic discomfort, and a burning sensation, to facilitate accurate postoperative reporting.

This study was initially designed as a prospective randomized controlled trial to evaluate the effect of an intravenous fixed-dose combination of 1000 mg acetaminophen and 300 mg ibuprofen on the incidence of CRBD. However, due to slower-than-anticipated patient recruitment, the study was terminated early and subsequently considered a pilot randomized controlled trial aimed at assessing feasibility and estimating the potential effect size. No formal interim analysis or predefined stopping rule was established before study initiation, and the trial was not terminated because of demonstrated efficacy or safety concerns. Early termination was reported to and acknowledged by the Institutional Review Board.

We enrolled patients aged 19–70 years with an American Society of Anesthesiologists (ASA) physical status I–III who were scheduled for urological surgery requiring urinary catheterization. A patient was excluded if any of the following criteria were met: (1) contraindication to acetaminophen or ibuprofen (e.g., history of hypersensitivity/anaphylaxis to acetaminophen or ibuprofen, gastrointestinal ulcers, severe renal impairment); (2) overactive bladder (OAB; urinary frequency > 3 times per night or > 8 times in 24 h); (3) bladder outlet and lower urinary tract obstruction; (4) benign prostatic hyperplasia; (5) active alcoholism; (6) neuropsychological disorder or cognitive impairment; and (7) cases with factors that were judged to have an impact on postoperative outcomes, such as combined surgery.

Patients were randomly allocated in a 1:1 ratio to either the control group or the intervention group using online randomization software (Research Randomizer 4.0; www.randomizer.org). Allocation assignments were placed in sequentially numbered, sealed, opaque envelopes to ensure allocation concealment. The envelopes were opened only after the patient’s arrival in the operating room by the attending anesthesiologist responsible for study drug administration (saline 100 mL for the control group and Maxigesic^®^ 1 vial [100 mL] for the intervention group). The study was conducted in a double-blind manner. The attending anesthesiologist who administered the study drug was not involved in postoperative outcome assessment, data collection, or statistical analysis. Patients, surgeons, and the anesthesiology resident responsible for postoperative assessments remained blinded to group allocation throughout the study period.

All patients fasted for at least 8 h prior to surgery and were transferred to the operating room without premedication. Standard monitoring included pulse oximetry, electrocardiography, non-invasive blood pressure monitoring, the Patient State Index (PSI) (SedLine^®^; Masimo Corp., Irvine, CA, USA), and neuromuscular train-of-four (TOF) acceleromyography on the adductor pollicis muscle. Anesthesia was induced with intravenous propofol (1.5–2 mg/kg) and fentanyl (1–2 μg/kg), followed by rocuronium (0.6 mg/kg) to facilitate endotracheal intubation. Anesthesia was maintained with N_2_O/O_2_ (1:1) and desflurane (3–8 vol% end-tidal concentration) to maintain the PSI at 25–50. The study drug was administered approximately 20 min before the anticipated end of surgery. Patients in the intervention group received an intravenous infusion of Maxigesic^®^ (1 vial), while those in the control group received 100 mL of normal saline. All infusions were administered over 15 min.

All patients underwent surgery in the lithotomy position. At the end of the surgery, urinary catheterization was performed using a Foley catheter lubricated with 2% lidocaine jelly (Instillagel^®^; Farco-Pharma GmbH, Cologne, Germany) and the balloons of the catheter were inflated with normal saline. The size of the Foley catheter and volume of the balloon were determined at the discretion of the urologist. After urinary catheterization, the patients were placed in the supine position. Subsequently, to reverse the neuromuscular block, sugammadex was administered intravenously according to the depth of the block, and the patients were transferred to the post-anesthetic care unit (PACU) after extubation. All patients were observed for at least 1 h in the PACU.

CRBD was assessed at 0, 1, 2, and 6 h after the patient arrived at the PACU. The severity of CRBD was evaluated using a 4-point scale (none = patient did not complain of any CRBD symptoms even when asked, such as urge to urinate or discomfort in the suprapubic region, mild = complaint of CRBD symptoms only on direct questioning, moderate = spontaneous complaint by the patient of CRBD symptoms without any behavioral responses [e.g., attempts to pull out the catheter, flailing limbs, or a loud vocal response], and severe = spontaneous complaint by the patient of CRBD symptoms with behavioral responses) [2,3,10] and an 11-point numeric rating scale (NRS, 0 = no catheter-related bladder discomfort, 10 = worst catheter-related bladder discomfort imaginable) by the anesthesiology resident. CRBD incidence was defined as the presence of mild or greater symptoms. At each assessment time point, CRBD was assessed first, and patients with moderate or severe symptoms then received intravenous tramadol (25–50 mg) as rescue medication.

The primary endpoint was the incidence of CRBD immediately after the patient arrived at the PACU. Secondary endpoints included the incidence of CRBD at 1, 2, and 6 h, the severity of CRBD (categorized as none, mild, moderate, or severe) at each time point, and the CRBD NRS assessed at PACU arrival and at 1, 2, and 6 h.

### Statistical Analyses

Based on prior data, the study was originally designed to enroll a total of 106 patients to detect a clinically meaningful difference in CRBD incidence. However, due to slower-than-expected recruitment, enrollment was discontinued early after enrolling 25 patients and the study was completed with a substantially smaller sample. Accordingly, the present trial should be considered a pilot randomized study. In light of the limited sample size, the analyses focused on estimation of effect size and confidence intervals rather than formal hypothesis testing.

SPSS Statistics software (ver. 27.0 for IBM Corp., Armonk, NY, USA) was used to perform the statistical analyses. The distribution of continuous variables was assessed with the Kolmogorov–Smirnov test; normally distributed variables were analyzed using Student’s *t*-test, and non-normally distributed variables were analyzed using the Mann–Whitney U-test. Categorical data were compared using the χ^2^ test, the χ^2^ test for trends (linear-by-linear association), or Fisher’s exact test as appropriate. Cohen’s effect sizes *d* and *h* were used to compare the continuous and categorical data, respectively. *p*-values are reported descriptively to support effect-size estimation and are not interpreted as evidence of statistical significance.

## 3. Results

**Figure 1:**
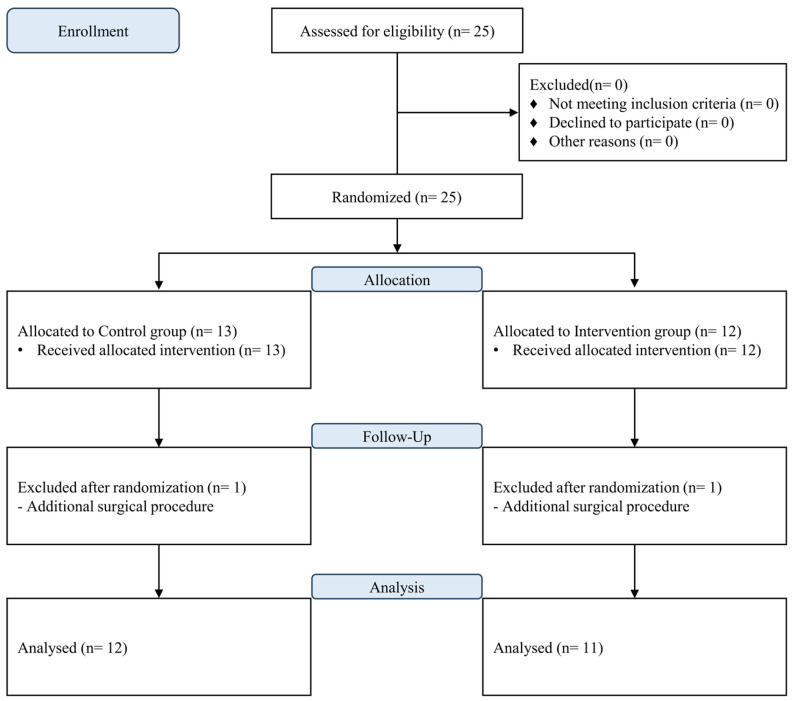
CONSORT flow diagram of patient enrollment, allocation, and analysis.

A total of 25 patients were enrolled and randomized. Two patients were excluded from the final analysis (one in each group) because they underwent additional intraoperative surgical procedures that were considered likely to affect postoperative CRBD assessment, resulting in 23 patients included in the outcome analysis (Figure 1). The characteristics and perioperative data of the patients in the two groups were comparable (Table 1).

**Figure 2:**
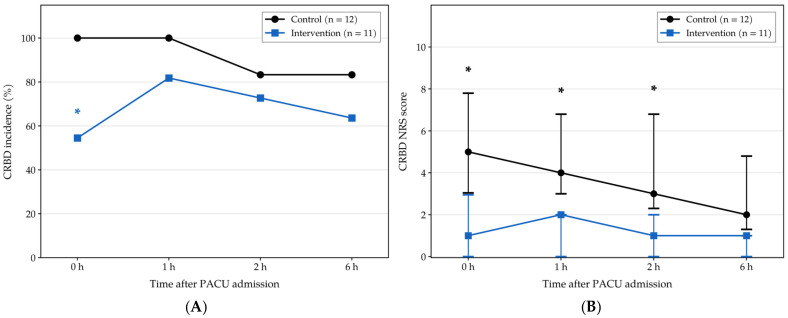
Postoperative catheter-related bladder discomfort (CRBD) outcomes over time in the control and intervention groups. (**A**) Incidence of CRBD after post-anesthetic care unit (PACU) arrival. (**B**) CRBD numeric rating scale (NRS) scores after PACU arrival. * *p* < 0.05 vs. control group. CRBD, catheter-related bladder discomfort; NRS, numeric rating scale; PACU, post-anesthetic care unit.

Data on the incidence and severity of postoperative CRBD are presented in Table 2. The incidence of CRBD upon arrival at the PACU, the predefined primary endpoint, was associated with a lower rate in the intervention group compared with the control group (54.5% vs. 100%; relative risk 0.545, 95% CI 0.34 to 0.87; absolute difference 45.5%, 98% CI 11.2% to 72.0%; effect size h 1.481, *p* = 0.014). However, no meaningful between-group differences in overall CRBD incidence were observed at 1, 2, or 6 h following PACU admission, suggesting that the observed benefit was primarily confined to the immediate postoperative period (Figure 2A).

Overall, CRBD severity appeared to differ between groups at PACU arrival (*p* = 0.002), but not at later postoperative time points. When severity categories were analyzed separately, the incidence of moderate CRBD was lower in the intervention group at 0 h (66.7% vs. 18.2%; absolute difference 48.5%, 98% CI 8.1% to 72.0%; effect size h 1.030, *p* = 0.036) and at 1 h (41.7% vs. 0%; absolute difference 41.7%, 98% CI 7.5% to 68.5%; effect size h 1.404, *p* = 0.037) following PACU admission. No between-group differences in moderate CRBD incidence were observed at 2 and 6 h postoperatively.

CRBD NRS scores were lower in the intervention group at 0, 1, and 2 h after patient arrival in the PACU (effect size d = 1.481, 1.070, and 0.929, respectively; *p* = 0.002, 0.011, and 0.023, respectively) (Figure 2B).

Fewer patients in the intervention group required rescue tramadol compared with the control group (9.1% vs. 41.7%; effect size h = 0.791), although this difference did not reach statistical significance (*p* = 0.155) (Table 2).

**Table 2:** Postoperative catheter-related bladder discomfort outcomes and rescue medication use.

	Control (*n* = 12)	Intervention (*n* = 11)	Difference (CI)	Effect Size *h* or *d*	*p*-Value
0 h					
Incidence	12 (100%)	6 (54.5%)	45.5% (11.2% to 72.0%)	1.481	0.014
Severity					0.002
Mild	3 (25.0%)	4 (36.4%)	−11.4% (−43.9% to 23.9%)	0.248	0.667
Moderate	8 (66.7%)	2 (18.2%)	48.5% (8.1% to 72.0%)	1.030	0.036
Severe	1 (8.3%)	0 (0%)	8.3% (−18.4% to 35.4%)	0.297	>0.999
CRBD NRS	5.0 (3.0, 7.8)	1.0 (0, 3.0)	3.5 (1.5 to 5.6)	1.524	0.002
1 h					
Incidence	12 (100%)	9 (81.8%)	18.2% (−9.4% to 47.7%)	0.881	0.217
Severity					0.068
Mild	6 (50%)	8 (72.7%)	−22.7% (−53.0% to 15.5%)	0.471	0.400
Moderate	5 (41.7%)	0 (0%)	41.7% (7.5% to 68.5%)	1.404	0.037
Severe	1 (8.3%)	1 (9.1%)	−0.8% (−30.2% to 27.3%)	0.028	>0.999
CRBD NRS	4.0 (3.0, 6.8)	2.0 (0, 2.0)	2.5 (0.5 to 4.5)	1.070	0.011
2 h					
Incidence	10 (83.3%)	8 (72.7%)	10.6% (−22.5% to 42.3%)	0.258	0.640
Severity					0.194
Mild	5 (41.7%)	7 (63.6%)	−22.0% (−52.8% to 16.7%)	0.442	0.414
Moderate	3 (25.0%)	0 (0%)	25% (−5.5% to 53.2%)	1.047	0.217
Severe	2 (16.7%)	1 (9.1%)	7.6% (−23.5% to 36.7%)	0.229	>0.999
CRBD NRS	3.0 (2.3, 6.8)	1.0 (0, 2.0)	2.3 (0.1 to 4.4)	0.929	0.023
6 h					
Incidence	10 (83.3%)	7 (63.6%)	19.7% (−15.5% to 50.4%)	0.453	0.371
Severity					0.315
Mild	8 (66.7%)	5 (45.5%)	21.2% (−17.1% to 52.3%)	0.431	0.414
Moderate	1 (8.3%)	2 (18.2%)	−9.9% (−40.2% to 20.2%)	0.297	0.590
Severe	1 (8.3%)	0 (0%)	8.3% (−18.4% to 35.4%)	0.584	>0.999
CRBD NRS	2.0 (1.3, 4.8)	1.0 (0, 1.0)	1.5 (−0.4 to 3.3)	0.698	0.069
Rescue Tramadol	5 (41.7%)	1 (9.1%)	32.6% (−3.8% to 60.0%)	0.791	0.155

Values are numbers (%) or median (Q1, Q3). CI: confidence interval; CRBD: catheter-related bladder discomfort; NRS: numeric rating scale (0 = no catheter-related bladder discomfort, 10 = worst catheter-related bladder discomfort imaginable); CRBD incidence was defined as ≥ mild symptoms. Differences represent absolute differences for categorical variables and mean differences for continuous variables. The 95% CIs are reported for incidence and CRBD NRS; the 98% CIs (Bonferroni-adjusted for three severity sub-categories: mild, moderate, severe) are reported for severity sub-categories.

## 4. Discussion

In this pilot randomized trial, administration of the acetaminophen–ibuprofen combination was associated with a lower incidence and reduced overall severity of CRBD at PACU arrival in patients undergoing urological surgery. This result corresponded to an absolute risk reduction of 45.5% and a number needed to treat of approximately three. CRBD NRS scores were also lower in the intervention group at 0, 1, and 2 h after PACU admission, although no clear between-group differences were observed at 6 h. In addition, fewer patients in the intervention group required rescue tramadol, although this finding did not reach statistical significance. Taken together, these findings provide preliminary evidence that the combination of acetaminophen and ibuprofen may contribute to a clinically meaningful reduction in early postoperative CRBD.

The observed reduction in early postoperative CRBD may be explained by the multimodal pharmacologic actions of the acetaminophen–ibuprofen combination. Mechanical irritation from urinary catheterization induces bladder discomfort through involuntary detrusor contraction and local inflammatory responses, including prostaglandin-mediated afferent sensitization [11,12]. Ibuprofen may attenuate peripheral inflammatory signaling through cyclooxygenase inhibition, whereas acetaminophen may modulate central nociceptive processing [13,14]. Accordingly, this combined regimen may reduce both peripheral and central components of catheter-related discomfort, which could explain the lower CRBD incidence and NRS scores observed during the early postoperative period in the present study.

The results of previous studies have shown that single-agent therapy with either intraoperative paracetamol [15] or NSAIDs such as ketorolac [16] can effectively reduce the incidence and severity of CRBD without significant adverse events. Our findings extend these observations by suggesting that a multimodal non-anticholinergic approach may provide clinically relevant early postoperative symptom reduction while potentially avoiding the adverse effects associated with antimuscarinic or gabapentinoid therapies commonly used for CRBD prevention [17,18,19,20].

The safety of the acetaminophen and ibuprofen combination is thus a key consideration, especially as it involves the simultaneous administration of two different pharmacological classes. While a fixed-dose combination could theoretically carry the side effect profiles of both agents, including gastrointestinal, renal, and hepatic concerns, extensive safety data from Cochrane reviews [9] and large-scale multi-center studies [8] demonstrate that short-term perioperative use of this combination is remarkably well tolerated. Specifically, the incidence of serious adverse events remains below 1%, with no significant increase in hepatic or renal toxicity compared to placebo [8,9]. In alignment with these established safety profiles, no serious drug-related adverse events, such as gastrointestinal bleeding or acute kidney injury, were observed in our study. These findings suggest that the multimodal benefits of this combination can be achieved safely, supporting its use as a reliable alternative for perioperative CRBD management.

From a clinical perspective, effective CRBD control is essential for optimizing perioperative outcomes. Beyond improving patient comfort, reducing CRBD helps mitigate emergence agitation and decreases the requirement for rescue medications, including opioids, which themselves may contribute to urinary retention and delayed discharge [21]. By ensuring a smoother transition from the PACU and facilitating early mobilization, this combination aligns with the core principles of ERAS protocols.

Several limitations of this study should be noted. First, the trial was terminated early due to recruitment difficulties, resulting in a smaller sample size than originally planned and consequently limited statistical power. Furthermore, the single-center design may limit the generalizability of the findings. Because the study was terminated early due to feasibility constraints without a predefined group sequential design or alpha adjustment, the findings should be interpreted cautiously and considered exploratory. Therefore, the present study should be regarded as a pilot randomized trial providing preliminary evidence regarding the potential effect of the intervention. Larger multicenter randomized studies are needed to validate these findings. Second, although catheter-related variables, including catheter size and balloon volume, were comparable between groups, these factors were not strictly standardized and may have contributed to residual confounding. In addition, other perioperative and intraoperative factors may have also contributed to variability in postoperative CRBD outcomes. Furthermore, rescue tramadol was administered to patients with moderate or severe CRBD for ethical reasons, which may have influenced subsequent assessment of CRBD severity and NRS scores. Because of the limited sample size of this pilot study, reliable subgroup or multivariable adjusted analyses were not performed. Third, we utilized a fixed-dose regimen, which precludes the determination of an optimal dose–response relationship. Moreover, the timing of administration may have influenced the results; the authors of future studies should therefore explore different dosing schedules. Despite these limitations, our findings may help inform sample size estimation and study design for future adequately powered randomized trials.

## 5. Conclusions

This pilot randomized trial provides preliminary evidence that the combination of 1000 mg acetaminophen and 300 mg ibuprofen may reduce early postoperative CRBD during the immediate recovery period following urological surgery, as reflected by lower CRBD incidence, reduced severity, and decreased CRBD NRS scores. However, the observed benefits were primarily limited to the early postoperative period and were not consistently sustained at later time points. In view of the small sample size and single-center design, these findings should be interpreted cautiously. Larger adequately powered multicenter randomized trials are required to confirm the clinical relevance of these preliminary observations.

## Figures and Tables

**Table 1:** Demographic and operative data.

	Control (*n* = 12)	Intervention (*n* = 11)	*p*-Value
Age, yr	54.2 ± 9.2	55.3 ± 9.7	0.783
Sex (male/female)	10/2	11/0	0.478
Weight, kg	71.1 ± 11.6	77.9 ± 10.4	0.158
Height, cm	167.5 ± 5.1	171.7 ± 8.4	0.155
BMI, kg/m^2^	25.3 ± 3.5	26.4 ± 3.0	0.433
ASA class (I/II/III)	0/10/2	1/7/3	0.941
Type of surgery			>0.999
Ureteroscopic litholapaxy	10 (83.3%)	10 (90.9%)	
TURBT	2 (16.7%)	1 (9.1%)	
Duration of surgery, min	37.5 (30.0, 98.8)	53.0 (30.0, 95.0)	0.786
Duration of anesthesia, min	65.0 (49.3, 123.8)	79.0 (51.0, 115.0)	0.525
Fluids (mL)	250.0 (200.0, 375.0)	300.0 (150.0, 500.0)	0.608
Urinary catheter size (Fr)			0.937
14/16/18/20/22	4/4/0/1/3	4/2/1/2/2	
Catheter balloon volume (mL)	5 (5, 5)	5 (5, 5)	0.963

Values are means ± standard deviation, numbers, numbers (%), or median (Q1, Q3). BMI: body mass index; ASA: American Society of Anesthesiologists; TURBT: transurethral resection of bladder tumors.

## Data Availability

The original contributions presented in this study are included in the article. Further inquiries can be directed to the corresponding author.

## Abbreviations

The following abbreviations are used in this manuscript:

CRBD	Catheter-related bladder discomfort
PACU	Post-anesthetic care unit
ERAS	Enhanced Recovery After Surgery
ASA	American Society of Anesthesiologists

## References

[1] Bai Y., Wang X., Li X., Pu C., Yuan H., Tang Y., Li J., Wei Q., Han P. (2015). Management of Catheter-Related Bladder Discomfort in Patients Who Underwent Elective Surgery. J. Endourol..

[2] Binhas M., Motamed C., Hawajri N., Yiou R., Marty J. (2011). Predictors of catheter-related bladder discomfort in the post-anaesthesia care unit. Ann. Fr. Anesth. Reanim..

[3] Kim H.C., Hong W.P., Lim Y.J., Park H.P. (2016). The effect of sevoflurane versus desflurane on postoperative catheter-related bladder discomfort in patients undergoing transurethral excision of a bladder tumour: A randomized controlled trial. Can. J. Anaesth..

[4] Markopoulos T., Katsimperis S., Lazarou L., Tzelves L., Mitsogiannis I., Papatsoris A., Skolarikos A., Varkarakis I. (2025). Catheter-Related Bladder Discomfort: Insights Into Pathophysiology, Clinical Impact, and Management. Cureus.

[5] Andersson K.E. (2004). Antimuscarinics for treatment of overactive bladder. Lancet Neurol..

[6] Andersson K.E., Arner A. (2004). Urinary bladder contraction and relaxation: Physiology and pathophysiology. Physiol. Rev..

[7] Daniels S.E., Playne R., Stanescu I., Zhang J., Gottlieb I.J., Atkinson H.C. (2019). Efficacy and Safety of an Intravenous Acetaminophen/Ibuprofen Fixed-dose Combination After Bunionectomy: A Randomized, Double-blind, Factorial, Placebo-controlled Trial. Clin. Ther..

[8] Gottlieb I.J., Gilchrist N., Carson S., Stanescu I., Atkinson H. (2021). Extending the safety profile of the post-operative administration of an intravenous acetaminophen/ibuprofen fixed dose combination: An open-label, multi-center, single arm, multiple dose study. Biomed. Pharmacother..

[9] Derry C.J., Derry S., Moore R.A. (2013). Single dose oral ibuprofen plus paracetamol (acetaminophen) for acute postoperative pain. Cochrane Database Syst. Rev..

[10] Cho S.A., Huh I., Lee S.J., Sung T.Y., Ku G.W., Cho C.K., Jee Y.S. (2022). Effects of dexamethasone on catheter-related bladder discomfort and emergence agitation: A prospective, randomized, controlled trial. Korean J. Anesthesiol..

[11] Giglio D., Tobin G. (2009). Muscarinic receptor subtypes in the lower urinary tract. Pharmacology.

[12] Daly D.M., Collins V.M., Chapple C.R., Grundy D. (2011). The afferent system and its role in lower urinary tract dysfunction. Curr. Opin. Urol..

[13] Burian M., Geisslinger G. (2005). COX-dependent mechanisms involved in the antinociceptive action of NSAIDs at central and peripheral sites. Pharmacol. Ther..

[14] Anderson B.J. (2008). Paracetamol (Acetaminophen): Mechanisms of action. Paediatr. Anaesth..

[15] Ergenoglu P., Akin S., Yalcin Cok O., Eker E., Kuzgunbay B., Turunc T., Aribogan A. (2012). Effect of intraoperative paracetamol on catheter-related bladder discomfort: A prospective, randomized, double-blind study. Curr. Ther. Res. Clin. Exp..

[16] Park J.Y., Hong J.H., Yu J., Kim D.H., Koh G.H., Lee S.A., Hwang J.H., Kong Y.G., Kim Y.K. (2019). Effect of Ketorolac on the Prevention of Postoperative Catheter-Related Bladder Discomfort in Patients Undergoing Robot-Assisted Laparoscopic Radical Prostatectomy: A Randomized, Double-Blinded, Placebo-Controlled Study. J. Clin. Med..

[17] Agarwal A., Dhiraaj S., Singhal V., Kapoor R., Tandon M. (2006). Comparison of efficacy of oxybutynin and tolterodine for prevention of catheter related bladder discomfort: A prospective, randomized, placebo-controlled, double-blind study. Br. J. Anaesth..

[18] Chapple C.R., Patroneva A., Raines S.R. (2006). Effect of an ATP-sensitive potassium channel opener in subjects with overactive bladder: A randomized, double-blind, placebo-controlled study (ZD0947IL/0004). Eur. Urol..

[19] Srivastava V.K., Agrawal S., Kadiyala V.N., Ahmed M., Sharma S., Kumar R. (2015). The efficacy of pregabalin for prevention of catheter-related bladder discomfort: A prospective, randomized, placebo-controlled double-blind study. J. Anesth..

[20] Verret M., Lauzier F., Zarychanski R., Perron C., Savard X., Pinard A.M., Leblanc G., Cossi M.J., Neveu X., Turgeon A.F. (2020). Perioperative Use of Gabapentinoids for the Management of Postoperative Acute Pain: A Systematic Review and Meta-analysis. Anesthesiology.

[21] Baldini G., Bagry H., Aprikian A., Carli F. (2009). Postoperative urinary retention: Anesthetic and perioperative considerations. Anesthesiology.

